# Terrain of taphonomy: how biogeographic variation affects decomposition and scavenger behaviour in two forensically significant habitats of Cape Town, South Africa

**DOI:** 10.1007/s00414-025-03470-w

**Published:** 2025-03-18

**Authors:** Kara Sierra Adams, Devin Alexander Finaughty, Victoria Elaine Gibbon

**Affiliations:** 1 Division of Clinical Anatomy and Biological Anthropology, Department of Human Biology, Faculty of Health Sciences, University of Cape Town, Cape Town, South Africa; 2 Human Variation and Identification Research Unit (HVIRU), Department of Anatomical Sciences, School of Biomedical Sciences, Faculty of Health Sciences, University of the Witwatersrand, Johannesburg, South Africa

**Keywords:** Post-mortem interval, Taphonomy, Scavenging, Western Cape, Decomposition, Ecology, Peri-urban environment, Open Cape Flats Dune Strandveld, Biogeographical region, Seasonality

## Abstract

In South Africa, high rates of unidentified human remains necessitate the establishment of regionally specific high resolution taphonomic data to facilitate accurate reconstruction of postmortem circumstances and timing, as well as identification. This study investigates the effects scavenging and environmental conditions on the decomposition process using porcine models as human analogs across two distinct forensic sites in Cape Town: a suburban site and a peri-urban site. Over four deployments (July 2021–January 2023), six clothed porcine bodies were placed at each site and monitored. Data collected included mass loss, scavenger activity (notably by the Cape grey mongoose *Galerella pulverulenta*), and environmental variables. Findings revealed that seasonal variations and habitat types had significant impacts on the rate and pattern of decomposition. Porcine bodies at the Medical Research Council site consistently decomposed faster than those at the University of Cape Town site due to the micro habitat differences documented between the two sites. This research underscores the importance of considering biogeographic variation and the displacement of vertebrate scavengers in urban settings, emphasising the need for careful site selection in decomposition research to better reflect some forensic urban scenarios. By replicating the locally prevalent medicolegal death scenario of a single clothed body, the study enhances understanding of postmortem processes in Cape Town and contributes to the refinement of methodologies for forensic taphonomy within specific ecological contexts.

## Introduction

In South Africa, forensic mortuaries and police face considerable challenges in identifying the remains of unknown decedents, with an estimated 10% of autopsied individuals remaining unidentified and eventually interred in pauper burials each year [1–4]. This high rate of unidentified remains—potentially numbering thousands annually—is influenced by several factors, including high mortality rates, migration, limited access to ante-mortem records, and the advanced decomposition or burning of remains [1, 4–7]. Forensic taphonomy, the study of post-mortem changes in human remains, is crucial to addressing these challenges, as it provides essential data for estimating the post-mortem interval (PMI) as well as interpreting scene evidence to reconstruct peri- and post-mortem events. In South Africa, as with other countries, the taphonomic process varies significantly depending on micro-habitat, generating regionally specific decomposition patterns influenced by diverse environmental factors, including climate, biogeoclimatic zones, and distinct local fauna and flora.

Carrion decomposition is primarily influenced by temperature, which regulates microbial activity, chemical reactions, and necrophagous arthropods, accelerating decay in warm conditions and slowing it in cold environments [8–12]. Moisture also plays a critical role, as water availability affects microbial proliferation, with humidity sometimes having a greater impact than temperature [13]. When moisture levels drop below 85%, decomposition accelerates, while higher levels can inhibit microbial activity [10, 14, 15]. Other factors, including insect activity, scavenging, soil pH, sun exposure, and rainfall, further shape the process [15]. However, decomposition rates vary significantly across biogeographic regions, deposition contexts, and seasonal conditions [15].

Vertebrate scavenging is a crucial but under-researched aspect of forensic taphonomy in South Africa. Studies from other regions indicate its substantial impact on remains. For instance, Komar [16] documented scavenging in 46% of 596 cases in New Mexico, while Ubelaker and Degaglia [17] identified evidence of animal scavenging in 15% of cases examined across North America. Similarly, Indra [18] reported scavenging activity in 31.8% of cases analysed in Switzerland. Young et al. [19], as cited in Indra et al. [20], observed a notably higher incidence, with 63% of cases showing scavenging evidence. The extent scavengers affect bodies depends on the species involved and the intensity of scavenging. Feeding activity often begins at orifices or preexisting wounds, granting access to internal tissues [21, 22]. Such behaviour can obscure critical forensic details related to the cause-of-death or produce lesions that may be misinterpreted as trauma [23]. Despite the significance of scavenging in forensic taphonomy, limited research exists on its effects in the South African context, where diverse scavenger species—including both vertebrates and invertebrates—may significantly alter decomposition processes.

Forensic taphonomic research conducted within the Western Cape Province of South Africa exemplifies the need for regionally specific studies. The province’s unique Mediterranean climate and the Fynbos biome, part of the Cape Floristic Kingdom, create distinct decomposition conditions that differ from other environments globally. This area has various microclimates influenced by its mountainous landscape and proximity to the ocean, further complicating decomposition patterns. Forensic Anthropology Cape Town (FACT), one of only two forensic anthropology laboratories in the province, works with the Forensic Pathology Service (FPS) and South African Police Service (SAPS) to aid identification efforts for skeletonised and decomposed bodies, but these cases require more robust, locally derived taphonomic data to improve the accuracy of efforts to identify decedents and determine what happened to them [5, 6].

The present study simulates a locally common medico-legal scenario in which a single clothed individual is discovered in a peri-urban or suburban area of the City of Cape Town – the province’s largest and most populous city [24]. By conducting field experiments in two different habitats, this research analyses how climate, scavenger activity, and other microhabitat factors affect decomposition specific to South Africa. Through advancing forensic taphonomy in South Africa, we aim to improve case outcomes, create baseline decomposition data, understand local and regional taphonomic nuances to support the critical goal of identifying unknown decedents in a way that honours their dignity and provides closure to their families.

## Materials and methods

**Fig. 1:**
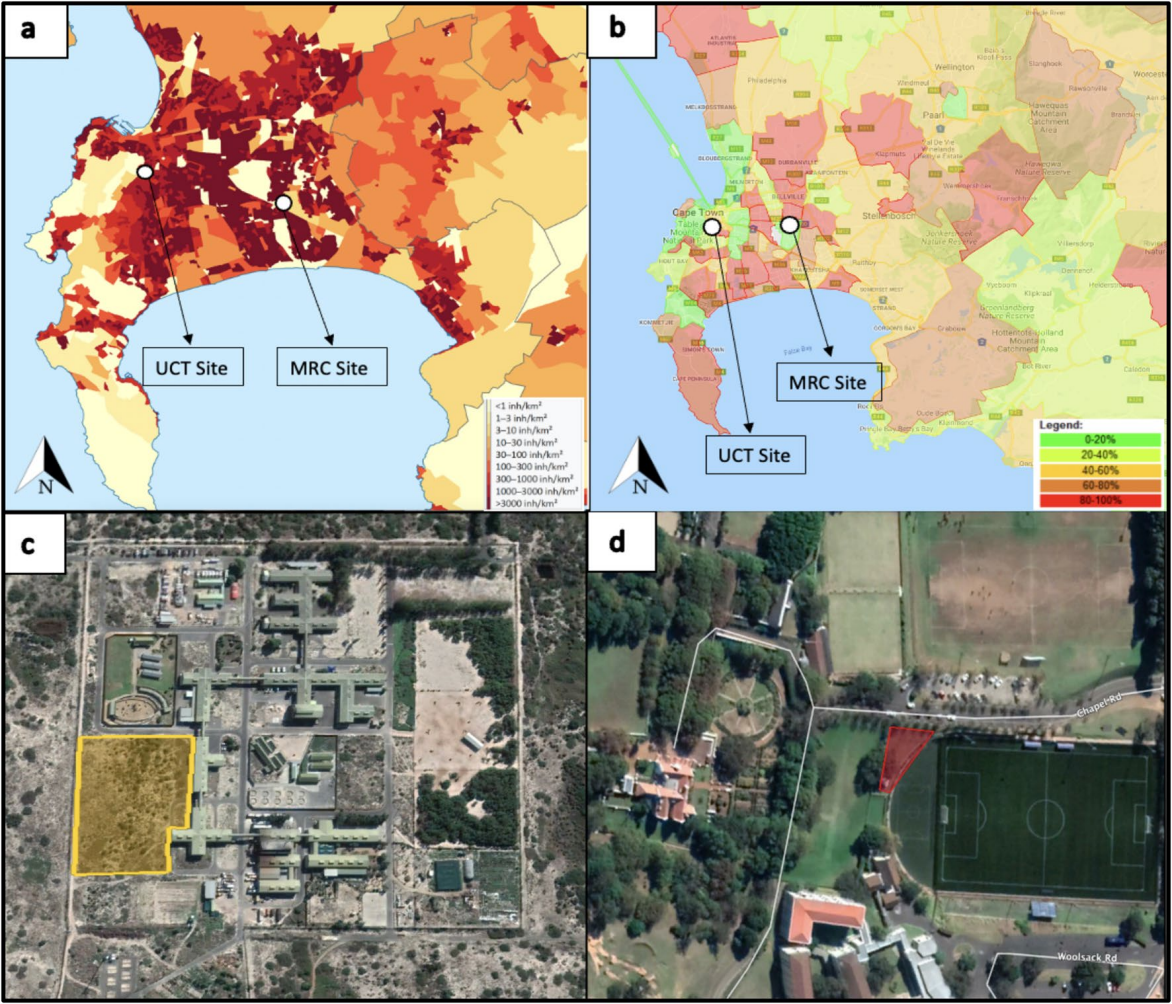
**a** Population density of Cape Town suburbs with the research sites indicated on the original map from the 2011 census at the “small area” level, 2015 (https://commons.wikimedia.org/wiki/File:Cape_Town_2011_population_density_map.svg). Note: the 2022 census indicates considerably population growth, though this graphic has not been updated by authorities as the census data have not been ratified at the time of writing; **b** Murder rate by suburb with research locations indicated on the original map Crime statistics South Africa, where the number of murders in each police precinct are heat-mapped according to their percentage contribution to overall murder statistics in South Africa (e.g., an 80–100% classification means that the police precinct in question is a top 20% contributor to murder numbers nationally) (http://www.crimestatssa.com); **c** MRC research site located in the suburb of Delft indicated on the map using Google Earth; **d** UCT research site located in the suburb of Rosebank indicated on the map using Google Earth [28]

This study was conducted at two forensically significant locations in Cape Town. Cape Flats Dune Strandveld (CFDS) and Cape Flats Sand Fynbos (CFSF) vegetation are endemic to the region and part of the Cape Floristic Region (CFR), a globally recognised biodiversity hotspot [25] they are also the most representative vegetation subtype(s) where a large portion of Cape Towns’ medico-legal death investigations come from [26, 27]. However, urbanisation has destroyed most of the CFSF vegetation; therefore, only the CFDS vegetation is represented in this study [25]. The first site was in a peri-urban CFDS habitat, and the second was in a suburban habitat. (Fig. 1). The research locations are described in further detail below. The two habitat types are classed as “MRC site” and “UCT site” (or variants thereof), respectively.

### South African Medical Research Council (MRC) site

The MRC research facility is in Delft, Cape Town, South Africa (Fig. 1). Delft is on the Cape Flats, a low-lying, flat area on the southeastern side of the City of Cape Town Metropole. The Cape Flats extend from the Boland Mountains in the east to the Table Mountain range in the west. Delft is a forensically significant region with a dense population of more than 13,000 inhabitants per square kilometre [29]. Additionally, Delft has a high murder rate, with 275 murders reported by the Delft police precinct in 2023/2024 alone – more than double the number of murders in the entire London Metropole in the same period [30].

The site is an outdoor peri-urban environment (UTM 17 T 283009.88 m E 6236344.50 m S), 3.64 acres in size and located on the west side of the MRC facility. The facility is enclosed by a multi-strand electric fence, easily accessed by animals who can pass through the gaps or dig beneath the fence through the soft sand substrate that dominates the area but deters access by people. The urban settlements of Delft have encroached up to the fence of the MRC, but the site remains adjacent to the Driftsands Nature Reserve, which has one of the largest intact remnants of CFDS vegetation. CFDS is characterised by flat, slightly undulating landscapes with sandy, nutrient-poor soils [25], and a large portion of medico-legal death investigation cases come from this vegetation type.

### UCT site

The UCT site is private land in Rosebank, Cape Town, South Africa, owned and managed by the University of Cape Town (Fig. 1). Rosebank has a population density of more than 4,500 inhabitants per square kilometre [29] and is a relatively low crime area compared to Delft, with only two murders reported for 2023/2024 [30]. It is forensically significant as research has found that body disposal in open fields and residential areas in Cape Town is common [5, 24, 31, 32].

The site was an outdoor suburban environment (UTM 17 T 265982.37 m E 6240100.73 m S), 0.09 acres in size, immediately surrounded by recreational sports fields. The site is enclosed by a fence that excludes almost all terrestrial animal species from accessing the land – not unlike those erected around most private properties in Cape Town (fence or brick wall). Rosebank is a suburban area with many residential homes and recreational fields and is located on the eastern slope of Table Mountain, though a motorway separates it from Table Mountain National Park. This location is representative of typical low-to-medium density suburban environments that make up the city.

### Study subjects

Six 60 kg pig (*Sus scrofa domesticus*) bodies were used as analogues for adult human bodies. The use of pigs as proxies for humans in taphonomic studies where the establishment of baseline data are concerned is widely accepted in contexts where the use of donated human bodies for such research is prohibited or otherwise not possible, like South Africa [33, 34]. The six clothed porcine bodies were deployed over four seasonal trials (in South Africa, winter is June to August and Summer is December to February), beginning in July 2021 and ending in March (Table 1). Due to various reasons beyond the researcher’s control, access to the MRC facility ceased after March 2022. As a result, the remaining deployments for this project took place solely at the UCT site. Previous research by Finaughty [35] had collected the baseline data for unclothed porcine bodies. The pigs were terminated by a single 0.22 calibre gunshot wound to the base of the brain. To ensure the animals’ welfare, this process was carried out by an industry professional with veterinary oversight, per the ethically approved protocol (FHS AEC 018_023). It is prudent to note that the pigs for this study were derived from feedlots where the pigs are destined for human consumption; thus, this research did not alter their final fate. Following termination, within an hour of death, the porcine bodies were lightly rinsed with water and sealed in body bags. Following the placement of the bodies in body bags, they were transported immediately to the two research sites without any refrigeration or freezing. In keeping with forensic realism, the bodies were deployed within two and a half hours of termination and clothed on site. Previous analysis of Forensic Anthropology Cape Town (FACT) case files conducted by Spies and colleagues [36] provided details regarding the most common clothing types. The clothing used included: underwear, cotton T-shirt, denim pants, and a leather belt in the summer and the addition of socks, shoes, and jerseys in the winter [36, 37]. To ensure the clothing fit accurately, alterations were made to accommodate the anatomical differences between humans and pigs. Spies [37] used measurements taken from a live 60 kg pig as a guide for tailoring the clothes: chest circumference was 87 cm; pelvis circumference was 81 cm; armpit height was 31 cm; shoulder height was 54 cm; groin height was 33 cm; thigh circumference was 45 cm; snout-to-tail length was 136 cm. Clothes were purchased in sizes to reflect these measurements. Tailoring alterations included shortening and tapering the pant legs and jersey arms according to the measurements detailed above [38].

**Table 1:** Pig body code, location, date and season for each deployment

Pig Code	Season	Year	Deployment Site
MRCW2021	Winter	2021	MRC
UCTW2021	Winter	2021	UCT
MRCS2022	Summer	2022	MRC
UCTS2022	Summer	2022	UCT
UCT2022	Winter	2022	UCT
UCT2023	Summer	2023	UCT

*MRC*  Medical Research Council, *UCT*  University of Cape Town

### Decomposition

**Fig. 2:**
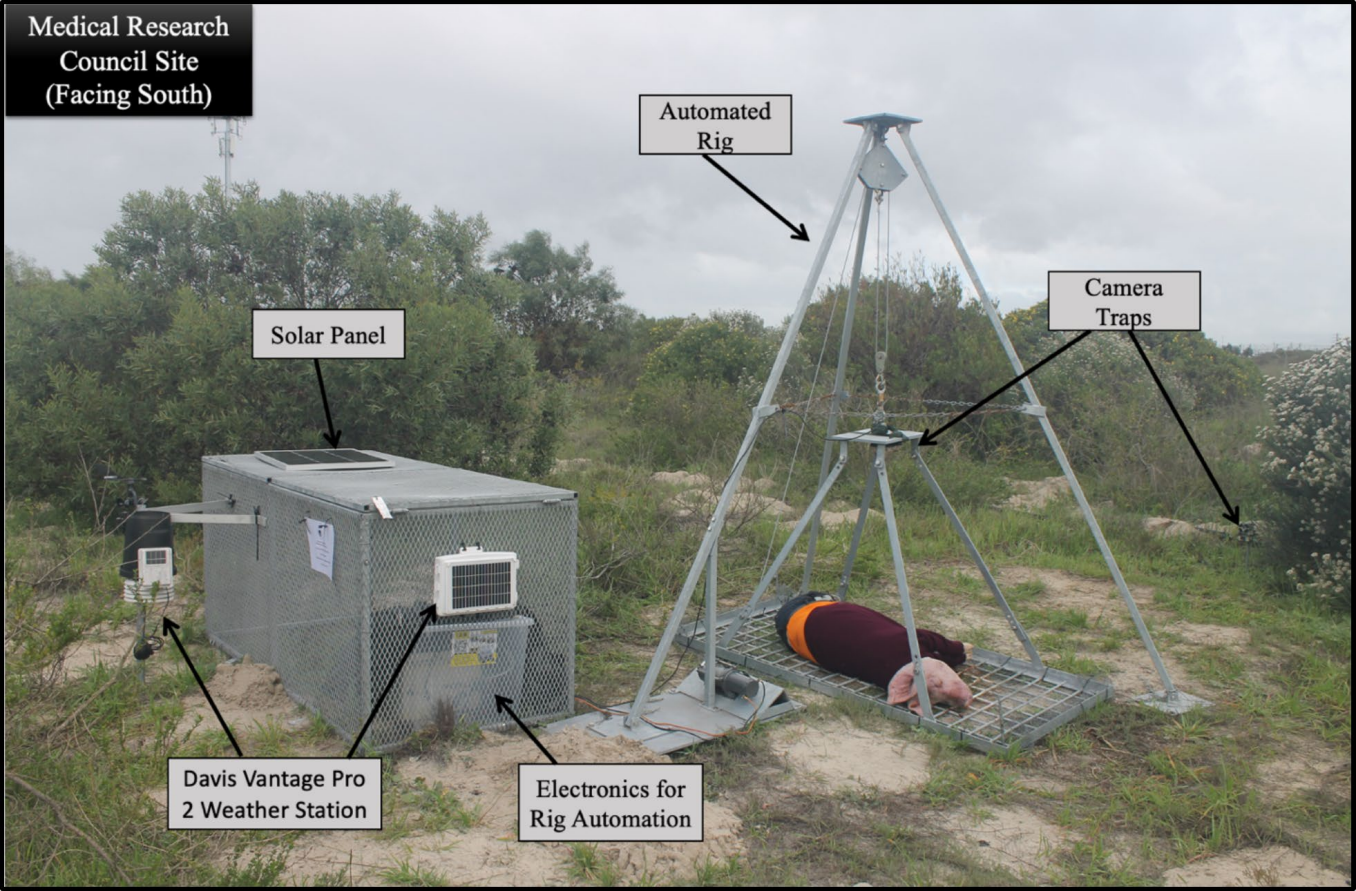
Medical research council site set up [28]

Decomposition rate (by proxy of body weight loss in kilograms over time) was determined using a solar-powered, automated, remotely accessible weighing apparatus [39, 40] (Fig. 2). Once every 24-h period (at midnight), the body would be lifted 10 cm off the ground for 20 s, weight readings would be obtained, and each lift generated an email sent to the researchers listing the body weight [28, 38]. Twenty readings were obtained during each lift and the average was used to account for any minor fluctuations or errors in the measurements. Decomposition progression was additionally visually assessed from photos obtained once per day from a standardised position directly over the body. The wide-angled trail camera was mounted above the body and was programmed to capture one photo at each hourly interval. Any missing data points were linearly interpolated in SPSS. This was accomplished using the visual criteria developed by Keough et al*.* [41] for the head and neck, and abdomen only, as clothing prevented visibility of the limbs. The preference for weight loss over time was adopted as a more standardised approach for quantifying decomposition, particularly given variations such as the presence of clothing on certain bodies [28, 38].

### Scavenging activity

Scavenging was constantly monitored throughout each deployment by a motion activated trail camera (Primos Proof Cam) that was positioned a few meters away pointed directly towards each body and was programmed to take three photos when triggered, rearming after 60 s. All camera trap data were analysed to document seasonal scavenging patterns using TimeLapse 2 [42]. Scavenging behaviour was categorised based on an adaptation of the criteria described by Dibner et al. [43]. All activity was categorised in one of three categories: “no contact” (the animal was visible but not on the grid of the rig); “close observation” (the animal was touching the weighing grid, but clearly not touching the body thereon); and “feeding/ direct contact” (the scavenger was either in direct contact with the body or had remains visibly in its mouth) [28, 38]. Visits were defined by the absence of the scavenger for 10 min or longer as described in previous research [43, 44].

## Results

### Winter (June to August)

A full breakdown of the winter weather variables is presented in Table 2. The 24-h mean temperature was 14.9 °C at the UCT site, which was 2.5 °C higher than at the MRC site. This trend was consistent for both daytime and nighttime mean temperatures. However, the difference in 24-h maximum temperature between the sites was not statistically significant, although the UCT site reached a maximum of 33.7 °C, 4.4 °C higher than the MRC site. In contrast, the minimum temperatures for 24-h, daytime, and nighttime periods were significantly different. While neither site experienced freezing temperatures (< 0 °C), the MRC site recorded a minimum temperature of 0 °C, which was 3.5 °C cooler than the lowest temperature recorded at the UCT site. Rainfall differences were not statistically significant, though the UCT site received considerably more rain (459.2 mm vs. 247.8 mm). Humidity was significantly different, with the MRC site experiencing higher levels (80.6% vs. 76.5%). Differences in windspeed were insignificant, with speeds of 2.3 km/h at MRC and 3.4 km/h at UCT. Solar radiation differed significantly, with the MRC site receiving 304.2 W/m^2^ compared to 252.3 W/m^2^ at UCT.

**Table 2:** Average weather variables recorded during all four cycles for 24 h, daytime, and night-time, along with habitat differences

Season	Period	Variable	Measure	MRC	UCT	Difference	*t*-statistic	*p*-value
Summer	24 h	Temperature (^o^C)	Max	42.3	38.4	3.9	−2.271	0.013
			Mean	23.0	22.6	0.4	−0.842	0.201
			Min	8.2	13.5	5.3	4.266	< 0.001
		Rainfall (mm)	Sum	13.2	13.7	0.5	−0.031	0.488
		Humidity (%)	Mean	70.9	71.1	0.2	0.133	0.447
		Windspeed (km/h)	Mean	2.4	1.0	1.4	−5.378	< 0.001
	Daytime	Temperature (^o^C)	Max	42.3	38.4	3.9	−2.271	0.013
			Mean	25.9	24.9	1.0	−1.495	0.069
			Min	8.2	13.5	5.3	3.643	< 0.001
		Rainfall (mm)	Sum	9.1	12.7	3.5	0.313	0.377
		Humidity (%)	Mean	63.0	64.6	1.6	0.693	0.245
		Windspeed (km/h)	Mean	3.0	1.2	1.8	−5.78	< 0.001
		Solar Radiation (W/m^2^)	Mean	469.3	381.3	88	−3.664	< 0.001
	Nighttime	Temperature (^o^C)	Max	28.7	27	1.7	−0.506	0.307
			Mean	18.5	19.3	0.8	1.625	0.054
			Min	8.2	13.7	5.5	3.559	< 0.001
		Rainfall (mm)	Sum	4.0	0.9	3.2	−1.669	0.049
		Humidity (%)	Mean	83.6	80.5	3.1	−2.011	0.024
		Windspeed (km/h)	Mean	1.5	0.7	0.8	−3.320	< 0.001
Winter	24 h	Temperature (^o^C)	Max	29.3	33.7	4.4	1.627	0.052
			Mean	12.5	14.9	2.5	7.725	< 0.001
			Min	0	3.6	3.6	11.235	< 0.001
		Rainfall (mm)	Sum	247.8	459.2	211.5	0.894	0.186
		Humidity (%)	Mean	80.6	76.5	4.1	−3.706	< 0.001
		Windspeed (km/h)	Mean	2.3	3.4	1.0	1.5	0.067
	Daytime	Temperature (^o^C)	Max	29.3	33.7	4.4	1.567	0.059
			Mean	15.1	16.5	1.4	4.183	< 0.001
			Min	0.4	3.6	3.2	9.863	< 0.001
		Rainfall (mm)	Sum	126.5	244.8	118.4	0.731	0.233
		Humidity (%)	Mean	73.3	72.1	1.2	−1.169	0.122
		Windspeed (km/h)	Mean	2.9	3.7	0.8	1.158	0.124
		Solar Radiation (W/m^2^)	Mean	304.2	252.3	51.9	−4.434	< 0.001
	Nighttime	Temperature (^o^C)	Max	19.7	24.6	4.5	5.822	< 0.001
			Mean	10.0	14.4	4.4	10.66	< 0.001
			Min	0.0	4.4	4.4	11.127	< 0.001
		Rainfall	Sum	121.3	214.5	93.2	0.664	0.254
		Humidity (%)	Mean	87.6	81.6	6.0	−5.930	< 0.001
		Windspeed (km/h)	Mean	1.8	3.0	1.2	1.797	0.037

*°C*  degrees Celsius, *mm*  millimetres, *% * percentage, *W/m*^2^  Watts per square meter, *km/h*  kilometres per hour; t- statistic from Welch two-sample t-test; significant differences (*p* ≤ 0.05) are denoted by bold type [28]

### Summer (December to February)

A full breakdown of the summer weather variables in presented in Table 2. Mean temperatures for 24-h, daytime, and nighttime periods were not statistically different between the two habitats. The 24-h mean temperature at the UCT site was 22.6 °C, 0.4 °C lower than at the MRC site. Conversely, the 24-h maximum temperature was significantly different; the UCT site reached a maximum of 38.4 °C, 3.9 °C lower than the MRC site, which peaked at 42.3 °C. Minimum temperatures across all periods showed statistically significant differences, with the MRC site recording the highest minimum temperature of 8.2 °C. Rainfall was similar and statistically insignificant, with the MRC site receiving 13.2 mm and UCT 13.7 mm. Humidity differences were also not significant, with the MRC site at 70.9% and the UCT site at 71.1%. Windspeed differences were significant, with the MRC site experiencing higher speeds (2.4 km/h vs. 1 km/h at UCT). Solar radiation was significantly different, with the MRC site receiving 469.3 W/m^2^ compared to 381.3 W/m^2^ at UCT.

### Scavenging activity

**Fig. 3:**
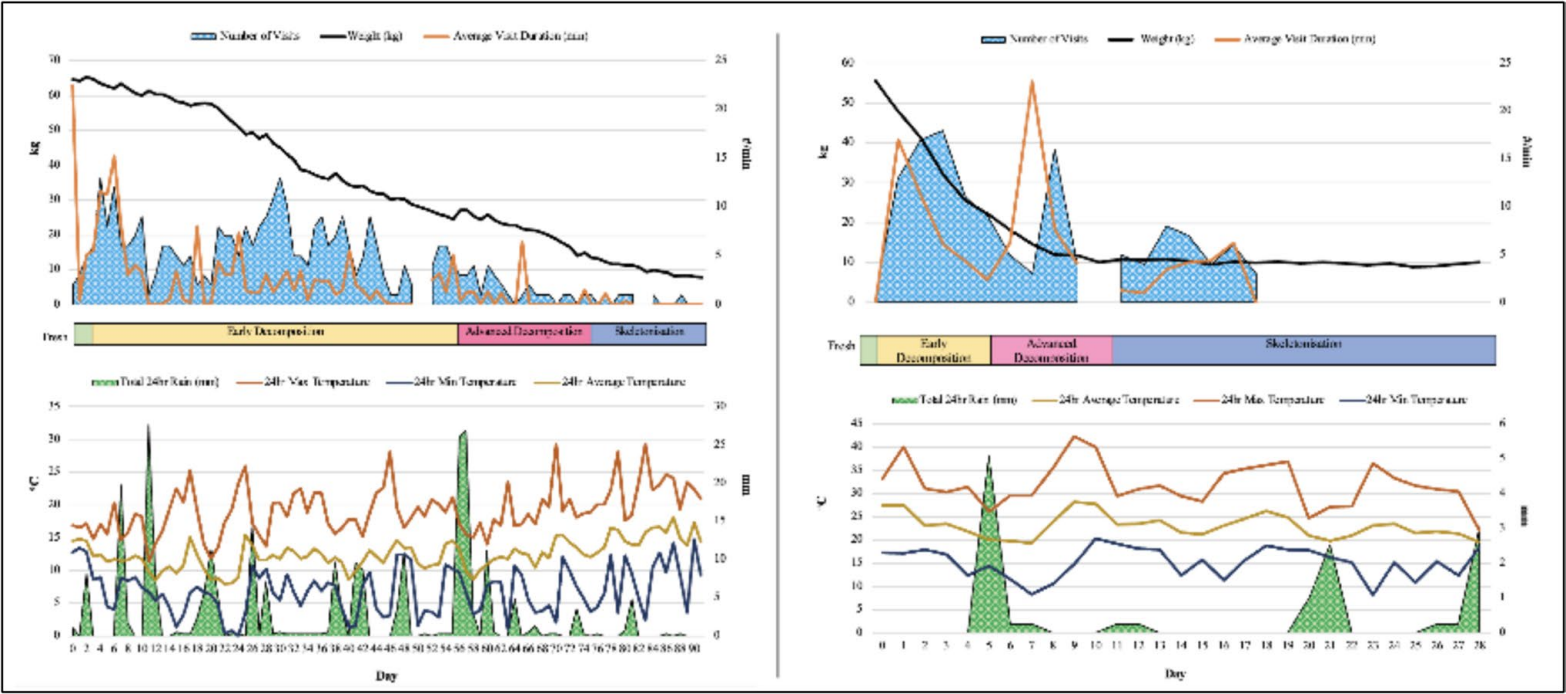
Cape grey mongoose scavenging activity recorded in the open habitat at the Medical Research council facility in Delft, Cape Town for Winter 2021 (left) and summer (right). ^o^C = degrees Celsius; mm = millimetre; kg = kilograms; # = number of visits; min = minutes. Adapted from Adams [28]

The Scavenging across all deployments only occurred at the MRC site. The MRC bodies were heavily scavenged in every deployment and the UCT ones were not scavenged at all. The Cape grey mongoose (*Galerella pulverulenta*), a small carnivorous mammal native to South Africa, was the only vertebrate scavenger documented, with activity varying by season. In winter, the mongoose made 351 visits (visits delineated by an absence of camera trap triggers for 10 min or longer) over a total duration of 21 h, 4 min, and 19 s, of which 9 h, 7 min, and 40 s involved direct feeding on the body. In summer, the mongoose made 135 visits totaling 14 h, 19 min, and 55 s, of which 6 h, 31 min, and 18 s involved direct feeding on the body. Winter feeding displayed a bimodal pattern, peaking during the fresh and middle decomposition stages, while summer feeding was restricted to the early decomposition phase. An overview of the scavenging activity can be seen in Fig. 3. For a comprehensive analysis of these scavenging patterns observed at the MRC during these deployments, see Adams, 2024 [38].

### Decomposition patterns

**Fig. 4:**
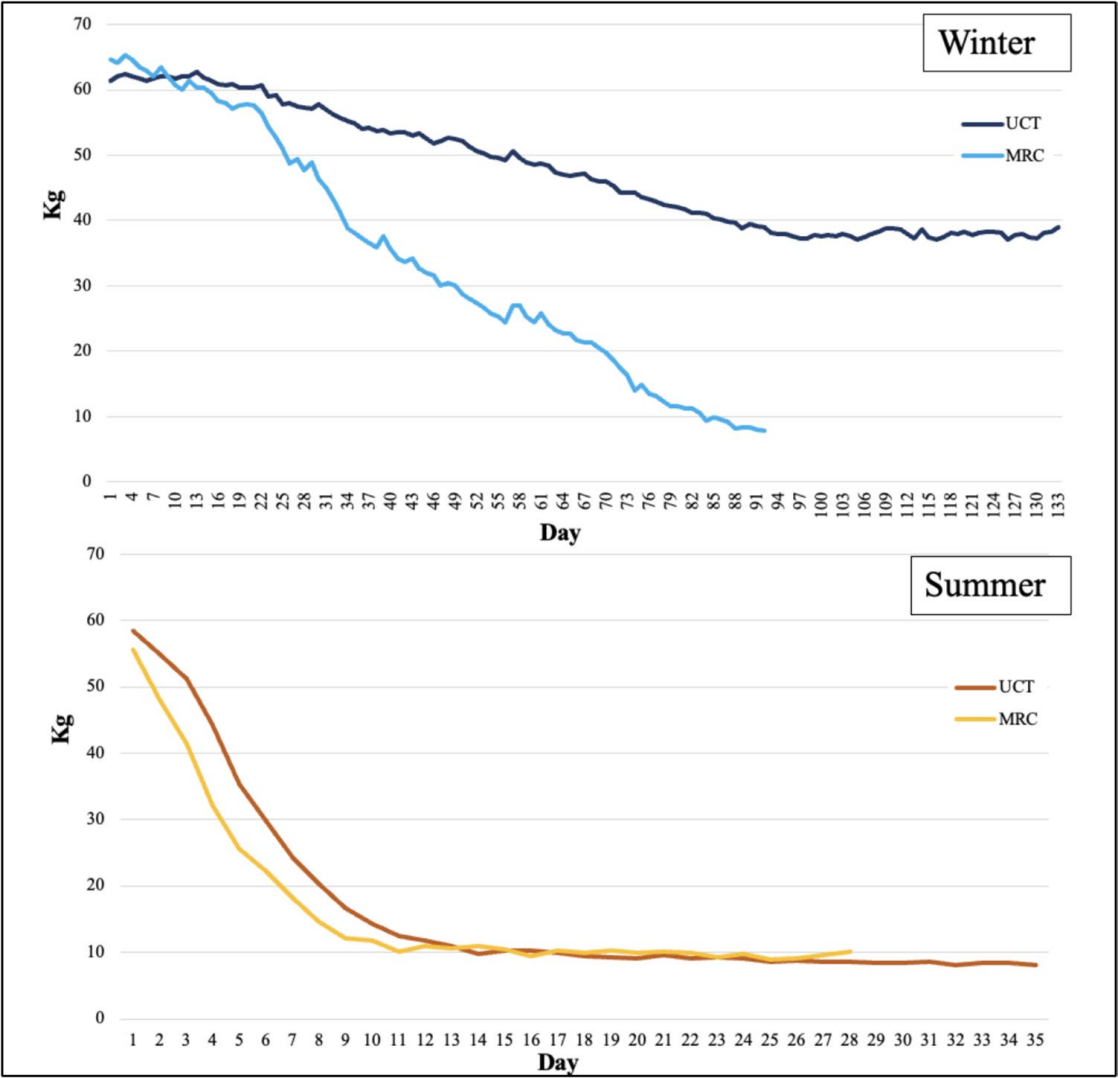
The seasonal differences in average body weight loss (kg) between the medical research council and University of Cape Town habitats [28]

The decomposition rate was quantified using weight loss over time. Data were wirelessly sent to the researcher, meaning the researcher only had to go to the site once a week. Missing data points were linearly interpolated in SPSS (29/430 days; 7% of the days). To benchmark the decomposition rate, weight loss percentage milestones were used: 25%, 50%, and 75%. These milestones were chosen as the bodies from this sample surpassed the 75% weight loss milestone, except those from the suburban UCT habitat during the winter months. The chosen quartiles were deemed appropriate because these two deployments did not reach 50% weight loss. An overview of the accumulated degree days (ADD) and time (in days) for each body to achieve the weight loss milestones are presented in Table 3. The seasonal differences in weight loss between the MRC and UCT habitats are shown in Fig. 4.

**Table 3:** ADD and time (in 24 h days) for each body to achieve the weight loss milestones

Body	Season	Deployment	25%WL ADD (Day)	50%WL ADD (Day)	75%WL ADD (Day)	End WL ADD (Day)	Total % lost
Single clothed bodies	Summer	UCT 2022	100.2 (3)	161.9 (6)	261.6 (10)	790.8 (34)	87%
		MRC 2022	78.1 (2)	123.5 (4)	206.7 (8)	648.2 (27)	82%
		UCT 2023	117.2 (4)	140.7 (5)	211.8 (8)	568.2 (24)	85%
	Winter	UCT 2021	872.8 (65)	-	-	1973.5 (132)	36%
		MRC 2021	330.4 (28)	508.0 (43)	864.3 (72)	1146.9 (91)	88%
		UCT 2022	951.9 (70)	-	-	1837.2 (122)	37%

*UCT * University of Cape Town, *MRC * Medical Research Council, *25%WL *25 percent weight loss, *50%WL* 50 percent weight loss, *75%WL* 75 percent weight loss, *End WL*  End weight loss, *Total % lost*  total percent lost, *ADD* accumulated degree days [28]

A comparative analysis of how the MRC and UCT bodies decomposed was required to assess the effect of season and habitat on decomposition (Table 4). There are existing various weather-related factors that are recognised for their impact on the decay rate. These differences may contribute to the variability observed between the two habitats.

**Table 4:** Kruskal–Wallis statistical tests for differences in accumulated degree days and time (24 h days) to weight loss milestones and stage of decomposition between UCT and MRC bodies, for A) summer and B) winter. Values bolded indicate a significant difference where *p* ≤ 0.05

A. Difference between habitat in summer.					
**Measure**	**MRC (1)**	**UCT (2)**	**Total (*n*=3)**	**H-stat (1)**	***p*-value**
ADD25%	78.2	108.69	93.38	1.5	.221
ADD50%	123.5	151.32	137.39	1.5	.221
ADD75%	206.7	236.7	221.68	1.5	.221
ADDend%	648.2	679.48	663.86	1.5	.221
Day25%	2	3.5	2.75	0.0	1.00
Day50%	4	5.5	4.75	1.5	.221
Day75%	8	9	8.5	0.5	.480
Dayend%	27	29	28	0.0	1.00
B. Difference between habitat in winter.					
**Measure**	**MRC (1)**	**UCT (2)**	**Total (*n*=3)**	**H-stat (1)**	***p*-value**
ADD25%	330.4	912.3	621.4	1.5	.221
ADD50%	508.0	-	-	-	-
ADD75%	864.0	-	-	-	-
ADDend%	1147.0	1905.7	1526.3	1.5	.221
Day25%	28	67.5	47.75	1.5	.221
Day50%	43	-	-	-	-
Day75%	72	-	-	-	-
Dayend%	91	127	109	1.5	.221

*ADD25%*  accumulated degree days to 25 percent weight loss, *ADD50%*  accumulated degree days to 50 percent weight loss, *ADD75%*  accumulated degree days to 75 percent weight loss, *ADDend% * accumulated degree days to final percent weight loss, *Day25%*  day to 25 percent weight loss, *Day50%*  day to 50 percent weight loss, *Day75%*  day to 75 percent weight loss, *Dayend% * day to final percent weight loss, *ADDfresh*  accumulated degree days to fresh stage, *ADDearly*  accumulated degree days to early decomposition stage, *ADDadv * accumulated degree days to advanced decomposition stage, *ADDskel* accumulated degree days to skeletonisation stage, *Dayfresh* days to fresh stage, *Dayearly*  days to early decomposition stage, *Dayadv* days to advanced decomposition stage, *Dayskel* days to skeletonisation stage [28]

### Weight loss

The winter MRC body took 28 days (ADD 330.4) to reach 25% weight loss, 43 days (ADD 508.0) to reach 50% weight loss, and 72 days (ADD 864.3) to reach 75% weight loss. At the end of the deployment period (91 days), the body had lost 88% of its initial weight. UCT bodies took 67.5 days (ADD 912.3) to reach 25% weight loss. These bodies did not reach the 50% or 75% weight loss milestones and entered a state of decompositional stasis. At the end of the deployment (127 days), the bodies had only lost 36.5% of their initial weight.

The summer MRC body took two days (ADD 78.1) to reach 25% weight loss, four days (ADD 123.5) to reach 50% weight loss, and eight days (ADD 206.7) to reach 75% weight loss. At the end of the deployment period (27 days), the body had lost 82% of its initial weight. UCT bodies took three and a half days (ADD 108.7) to reach 25% weight loss, five and a half days (ADD 151.3) to reach 50% weight loss, and nine days (ADD 236.7) to get to 75% weight loss. At the end of the deployment period (29 days), they had lost 86% of their initial weight.

**Fig. 5:**
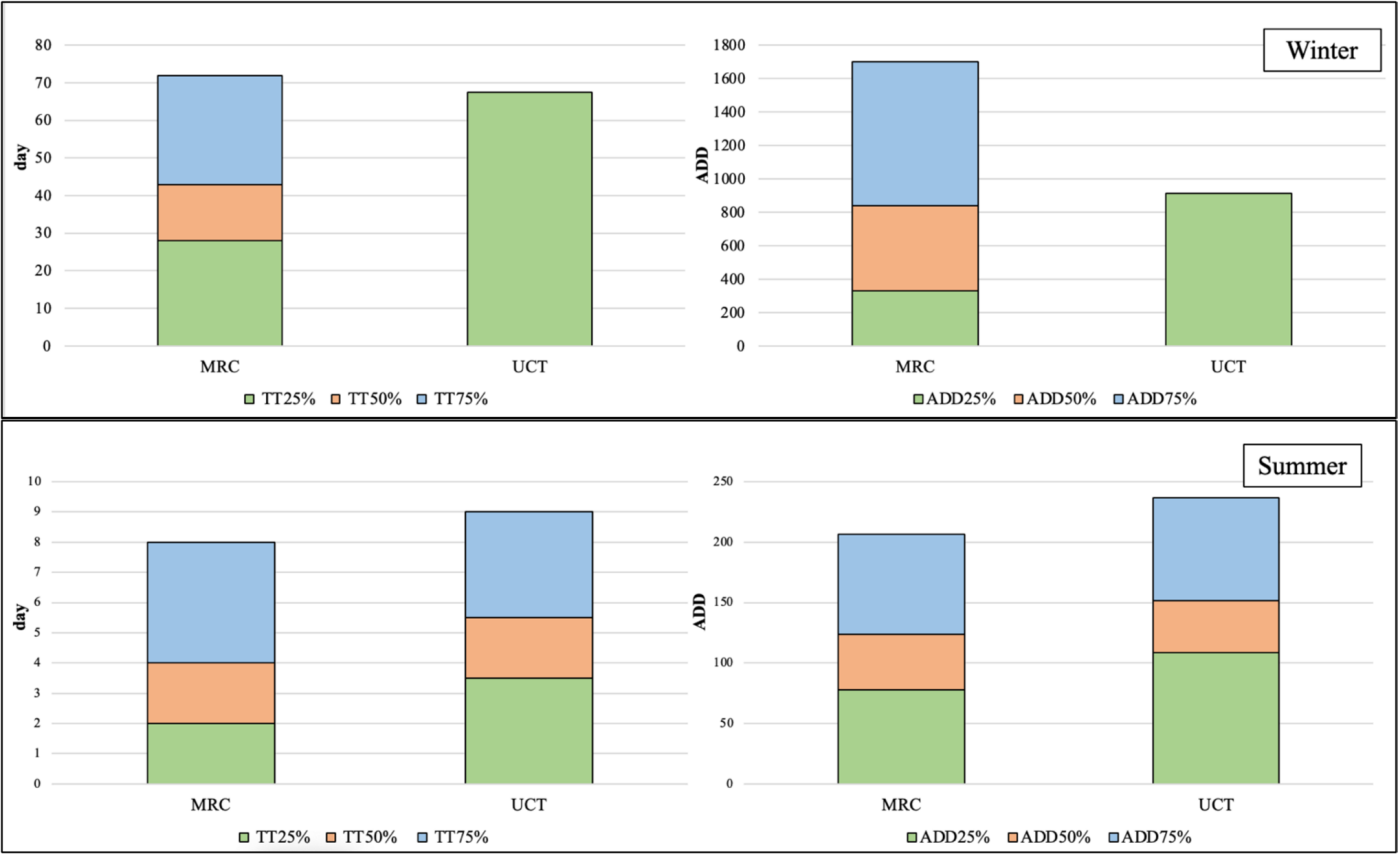
The mean number of days (left) and accumulated degree days (right) to reach weight loss percentiles for clothed and unclothed bodies in winter (top) and summer (bottom). TT25% = days to 25 percent weight loss; TT50% = days to 50 percent weight loss; TT75% = days to 75 percent weight loss; ADD25% = accumulated degree days to 25 percent weight loss; ADD50% = accumulated degree days to 50 percent weight loss; ADD75% = accumulated degree days to 75 percent weight loss; UCT = University of Cape Town; MRC = Medical Research Council [28]

Overall, the summer bodies lost weight quicker than the winter bodies, regardless of habitat. In both seasonal deployments, the MRC body progressed through the mass loss milestones faster than the UCT bodies. The weight loss data are presented in Table 5 and as stacked bar graphs in Fig. 5.

**Table 5:** Accumulated degree days and time (in 24 h days) to weight loss milestones

Season	Habitat	25%WL ADD (Day)	50%WL ADD (Day)	75%WL ADD (Day)	End WL ADD (Day)	Total % Lost
Summer	UCT	108.7 (3.5)	151.3 (5.5)	236.7(9)	679.5 (29)	86%
	MRC	78.1 (2)	123.5 (4)	206.7 (8)	648.2 (27)	82%
Winter	UCT	912.3 (67.5)	-	-	1905.7 (127)	36.5%
	MRC	330.4 (28)	508.0 (43)	864.3 (72)	1147.0 (91)	88%

*UCT* University of Cape Town, *MRC* Medical Research Council, *25%WL* 25 percent weight loss, *50%WL* 50 percent weight loss, *75%WL* 75 percent weight loss, *End WL*  End weight loss, *Total % lost* total percent lost, *ADD* accumulated degree days [28]

## Discussion

The MRC and UCT habitats experienced different rates of decomposition due to two main factors: first, the presence of the Cape grey mongoose – the sole vertebrate scavenger documented—and second, slight differences in their micro-climates. In winter, the MRC body reached > 80% weight loss after 91 days (ADD 1146.95); conversely, after 127 days (ADD 1905.73), the UCT bodies’ weight loss mean had not progressed past 36.5% of the original average weight of the bodies. In summer, the difference in the decomposition rate was less pronounced. The MRC body only reached each of the weight loss milestones one day before the UCT bodies did on average.

### Scavenging

In this study, the Cape grey mongoose did not feed at the site of the gunshot wound, possibly due to the wound’s small diameter (0.22 inches) and its location in an area with minimal soft tissue [36, 37]. Spies [37] also noted that this scavenger species focused on consuming soft tissues while avoiding desiccated areas or exposed bone. Additionally, the study corroborates previous findings that the Cape grey mongoose does not create new, noticeable bone lesions during feeding. Trauma, while not specifically investigated here, has been cited by other researchers as a factor influencing decomposition and can facilitate bacterial colonisation and insect oviposition, accelerating decomposition [11, 45, 46]. Contrarily, studies by Breitmeier et al. [47], Kelly [48], and Cross & Simmons [22] suggest that trauma neither accelerates decomposition nor attracts oviposition preferentially.

Some scavengers are also known to disarticulate remains, scattering skeletal elements and complicating recovery efforts [49–54]. However, no disarticulation was observed in the current study. Spies et al. [55] documented mongoose-induced scattering during the skeletonisation phase, with bones displaced up to 12.67 m into dense undergrowth. This behaviour occurred only once in experiments using 20 kg porcine bodies and was absent in subsequent studies involving 60 kg bodies [37]. The larger size and robustness of the bodies in this study likely reduced the potential for disarticulation.

Scavenging presents challenges to estimating the postmortem interval by accelerating decomposition rates and altering expected patterns of decay [20]. Scavenging by the Cape grey mongoose significantly increased decomposition rates during both seasonal deployments in the MRC habitat. These findings align with previous observations by Spies et al. [36, 56, 56] and Spies [37], which demonstrated that scavenging in thicketed habitats accelerates decay. Similar patterns have been observed with other small mammals, such as the striped skunk, which can alter PMI estimates by expediting skeletonisation and mummification [57]. Raccoons also disrupt decomposition by altering insect activity and promoting mummification [58]. Both species exhibit distinctive feeding behaviours, such as creating small holes in the skin to access tissue, leaving behind desiccated layers [58–65]. In the Midwestern USA, opossums significantly accelerated decomposition by consuming internal organs and soft tissues, followed by skeletal muscle, integument, and bone [66].

In the current study, entomological data were not collected or analysed. While daily photographs provide detailed visual documentation necessary for decomposition analysis, they do not offer sufficient detail to comment on insect activity. However, a previous study conducted at the MRC by Finaughty [35], examined entomological aspects and established baseline data on the role of necrophagous insects in decomposition in the habitat under study. This study identified 15 forensically significant taxa, including blow flies (e.g., *Chrysomya albiceps*, *Lucilia sericata*) and beetles like *Necrobia rufipes* and *Dermestes maculatus*. Species richness and abundance generally increased with decomposition progression, varying by season and habitat.

These findings emphasise the considerable impact of small vertebrate scavengers on decomposition across diverse biogeoclimatic regions. The discrepancy between scavenging activity at the two sites raises critical questions about the role of habitat type and vertebrate scavenger access in decomposition. The absence of scavengers at the UCT site, possibly due to its fencing and suburban environment, suggests that environmental barriers can significantly impact decomposition rates. Continued research is essential to quantify their effects and incorporate scavenging behaviour into PMI estimation methodologies.

### Climatic influence

There were slight differences in the set-up of the two habitats during the field deployments. While the MRC habitat was set up in a peri-urban area, the UCT site was in a suburban area. It required the addition of shade cloth around the perimeter of the body as a condition of the ethics to prevent visibility for any passersby. While there was no obstruction to the UCT body, the cloth shaded portions of the body at times when the sun was not directly overhead. While the effect of shade versus sun was not within the scope of this study, it was an unintentional confounding factor that requires mentioning. While differences are bound to exist, solar radiation is directly affected by shade cloth as well as the proximity of the UCT site to Table Mountain; the MRC site will experience sunlight for longer in the day as the mountain’s shadow cast by the setting sun will shade the UCT body first. This is observed in the overall lower solar radiation in summer (469.4 W/m^2^ at the MRC and 381.3 W/m^2^ at the UCT) and winter (304.2 W/m^2^ at the MRC and 252.3 W/m^2^ at the UCT). Few international studies have explored the difference in decomposition between shade and sun-deployed bodies. The UCT bodies that experienced occasional shade appeared to decompose slower than the complete sun bodies. This is consistent with Rhine and Dawson [67], Komar and Beattie [68], Shean and colleagues [69], and Majola and colleagues [70], who found that sun-exposed bodies decomposed faster, and full-shade and partial-shade delayed the overall decay rates. Comparatively, Sharonowski and colleagues [71] noted few differences in decomposition between sun-exposed and shaded bodies. The habitat variation affected species diversity, with the sun-exposed bodies attracting a greater diversity and number of species. Similarly, in Australia, Fitzgerald and Oxenham [72] found that sunlight had an insignificant effect on decomposition.

Our findings were inconsistent with the data reported by Cockle and Bell [73], who noted that complete shade bodies demonstrated faster decomposition than those in full sun. The differences between our study and the findings of Cockle and Bell [73] may stem from several key factors. First, retrospective case data inherently include a wide range of environmental and taphonomic variables—such as burial depth, seasonality, and scavenger presence—which are difficult to control and standardise. Additionally, the Canadian cases analysed by Cockle and Bell spanned multiple biogeographic regions, including temperate forests, boreal zones, and coastal areas. In contrast, our study was conducted under controlled conditions, allowing us to isolate the effects of specific microhabitat differences. They also did not support the findings of Bass [45], whose observations at the taphonomy facility in Knoxville, Tennessee, suggest that high heat and humidity in shaded conditions are optimal for maggot infestation and overall decomposition. The disparity between our findings and those of Bass [45] may be attributed to the distinct climatic and geographic differences between the continental/inland environment of Tennessee and the temperate coastal environment of Cape Town. Additionally, differences in scavenger species and activity may have played a significant role in altering decomposition rates across these two regions.

The UCT bodies consistently decomposed slower than the MRC bodies. This research suggests that this was more likely due to differences in vertebrate scavenging activity compared to differences in micro-climate between the two habitats. Additionally, some climactic variables, such as solar radiation, were found to be significantly different between habitats and could, therefore, also contribute to differences in decomposition rate between the sites.

### Study limitations

Limitations include the use of porcine models (at least as far as being able to abstract the results directly to human forensic cases), a small sample size, and access issues that restricted some deployments to only the UCT site. While the study provides baseline data on decomposition patterns, the small sample limits the statistical robustness, furthering the need for future research with additional bodies deployed simultaneously in habitat replicates over extended periods. Additionally, the technology used for data collection may have influenced scavenging behaviours, and the impact of the weigh-grid scale apparatus on decomposition remains uncertain.

## Conclusions

This study provides essential insights into how biogeographic variation impacts decomposition and scavenger activity in Cape Town’s habitats. Employing field experiments with porcine models has underscored the significant influence of environmental conditions and animal scavenging on the decomposition process of large carrion, emphasising the faster decay observed at the peri-urban MRC site compared to the suburban UCT site. Furthermore, this research demonstrates the importance of conducting taphonomic studies in locally forensically relevant environments, generating useful baseline data. One of the key findings of this study is the demonstration of how absence of vertebrate scavengers in some settings can significantly alter the rate and pattern of decomposition. This highlights two critical considerations: firstly, the need for more deliberate selection of decomposition research sites to better represent forensic scenarios, and secondly, the importance of paying closer attention to small vertebrate scavengers in urban settings. The data generated contributes to our taphonomic baseline information, ultimately enhancing forensic investigations in the Western Cape by providing a more comprehensive understanding of large carrion decomposition in Cape Town. These findings underscore the need for continued research to deepen our understanding and refine methodologies for interpreting forensic taphonomy within specific ecological contexts, particularly focusing on the role and impact of forensically significant vertebrate scavengers in urban environments.

## Data Availability

The data that support the findings of this study are available from the corresponding author, [VG], upon reasonable request.
